# Combining epidemiology with basic biology of sand flies, parasites, and hosts to inform leishmaniasis transmission dynamics and control

**DOI:** 10.1371/journal.ppat.1006571

**Published:** 2017-10-19

**Authors:** Orin Courtenay, Nathan C. Peters, Matthew E. Rogers, Caryn Bern

**Affiliations:** 1 School of Life Sciences, University of Warwick, Coventry, United Kingdom; 2 Zeeman Institute, University of Warwick, Coventry, United Kingdom; 3 Snyder Institute for Chronic Diseases, University of Calgary, Calgary, Alberta, Canada; 4 Department of Disease Control, London School of Hygiene and Tropical Medicine, London, United Kingdom; 5 Department of Epidemiology and Biostatistics, School of Medicine, University of California San Francisco, San Francisco, California, United States of America; Boston College, UNITED STATES

## Abstract

Quantitation of the nonlinear heterogeneities in *Leishmania* parasites, sand fly vectors, and mammalian host relationships provides insights to better understand leishmanial transmission epidemiology towards improving its control. The parasite manipulates the sand fly via production of promastigote secretory gel (PSG), leading to the “blocked sand fly” phenotype, persistent feeding attempts, and feeding on multiple hosts. PSG is injected into the mammalian host with the parasite and promotes the establishment of infection. Animal models demonstrate that sand flies with the highest parasite loads and percent metacyclic promastigotes transmit more parasites with greater frequency, resulting in higher load infections that are more likely to be both symptomatic and efficient reservoirs. The existence of mammalian and sand fly “super-spreaders” provides a biological basis for the spatial and temporal clustering of clinical leishmanial disease. Sand fly blood-feeding behavior will determine the efficacies of indoor residual spraying, topical insecticides, and bed nets. Interventions need to have sufficient coverage to include transmission hot spots, especially in the absence of field tools to assess infectiousness. Interventions that reduce sand fly densities in the absence of elimination could have negative consequences, for example, by interfering with partial immunity conferred by exposure to sand fly saliva. A deeper understanding of both sand fly and host biology and behavior is essential to ensuring effectiveness of vector interventions.

## Author summary

We review recent research that sheds light on the quantitative biology of leishmanial transmission between sand flies and mammalian hosts and use these insights to better understand transmission, the observed epidemiology of the disease, and their implications in choice of control strategy. Using animal models, we show how the parasite-induced processes manipulate sand fly blood-feeding behavior and the infectious metacyclic dose to promote host infection and to differentially regulate the onward transmission potential of individual vectors and hosts. The existence of subpopulations of mammalian and sand fly “super-spreaders” provides a biological basis for the spatial and temporal clustering of clinical leishmanial disease. While tools are unavailable to distinguish these individuals in mixed populations, blanket interventions will be necessary to ensure inclusion of transmission hot spots. Interventions that reduce sand fly densities without elimination could interfere with vector—host dynamics and conferred partial immunity to host populations.

## Introduction

In the Indian subcontinent, an effort to eliminate anthroponotic visceral leishmaniasis (VL) has been underway for a decade, and the incidence of the most severe clinical form, kala-azar, is at its lowest levels in 45 years. The program appears to be on track to “eliminate VL as a public health problem” by 2020 (defined as kala-azar incidence <1 case per 10,000 population) [1]. However, true elimination of transmission will be more elusive and requires a deeper understanding of the biology underlying transmission and disease. Substantial VL and cutaneous leishmaniasis (CL) burdens occur in many other continents, but the transmission dynamics and reservoir hosts differ, and development of tools for control and elimination are less advanced than in South Asia [2]. In this article, we review recent research that sheds light on the quantitative biology of leishmanial transmission between sand flies and mammalian hosts and use these insights to better understand observed patterns of VL and CL transmission and disease.

## Leishmaniasis clusters in time and space

**Fig 1:**
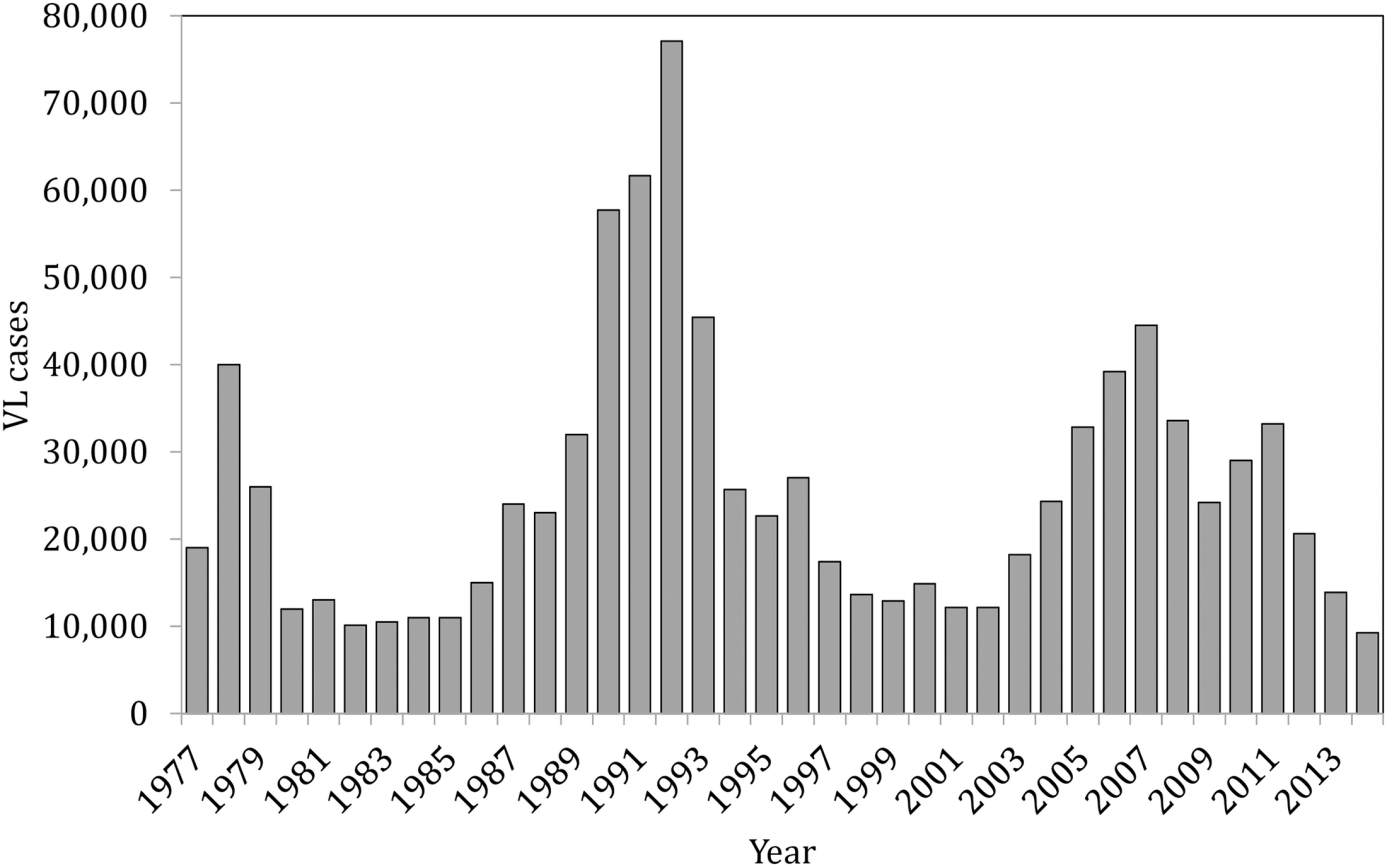
Reported cases of visceral leishmaniasis in India from 1977 to 2014. Data 1977–1985 are from Bihar only; data from 1986 onward include all reported cases in India. *Source of data: Ministry of Health and Family Welfare, Government of India, as published in* [17, 18].

Since VL was first studied in India nearly a century ago, investigators have observed incidence cycles that rise and fall with a slow periodicity [3]. Cycles have been documented in India, Bangladesh, Sudan, and Brazil [4–8]. A single cycle tends to last 5 to 15 years, with interepidemic intervals of 10 to 30 years [4, 5, 8]. At a regional level, climatic factors may contribute to these periodic cycles [9]. In a community, the fall in incidence after several peak years is thought to result from the buildup of herd immunity, with new epidemic onset occurring when a sufficient number of susceptible residents have accumulated through births and/or in-migration [3, 6]. The current best measure of protective immunity is the leishmanin skin test (LST), which reflects durable cell-mediated immunity. Individuals with a positive LST have more than 95% lower risk of kala-azar compared to those with negative LST, and the age-related rise in positive LST prevalence parallels an age-related decrease in disease risk [10–12]. In contrast, exposure to infective sand flies is variably age dependent [12, 13]. A fall in the average age of kala-azar patients may be observed as an epidemic matures [14]. The level of herd immunity required to end an epidemic cycle and the time to reach this level likely vary depending on parasite virulence, transmission intensity, vector exposure patterns, and host factors such as nutritional status and access to treatment. Interventions such as vector control and rapid case detection and treatment may alter the cycle but have not been shown to eliminate the periodicity. Intensive blanket DDT spraying during the malaria eradication program of the 1950s–1960s prolonged the interepidemic period in the Indian subcontinent, but since the resurgence in the 1970s, there have been 3 typical epidemic cycles in India and 2 in Bangladesh (Fig 1) [6, 15–18].

Periodic epidemic cycles represent clustering in time; the second major characteristic of VL epidemiology is clustering in space. On a global scale, VL is highly clustered, with 90% of the disease burden occurring in relatively few states or districts within just 6 countries: India, Bangladesh, Sudan, South Sudan, Brazil, and Ethiopia [4]. At smaller spatial scales, VL-affected communities and census tracts cluster in space and time [19, 20]. At the most local scale, strong clustering is seen at the household and near-neighbor levels [20–23]. Small-scale clustering is most marked early in an epidemic cycle when most community residents are susceptible and tends to disappear as the prevalence of immunity rises [21]. Clustering is likely due to macro- and microenvironmental conditions that promote sand fly breeding, survival, and aggregation, including proximity to reservoir and nonreservoir blood sources (humans, dogs, or other animals) [9, 19, 20, 24]. Sand fly aggregations are mediated by complex host—sand fly interactions (e.g., [25]) including sex-aggregation pheromones released by males of some species [26, 27], host kairomones, or plant phytochemical attractants [28]. Temporal clustering of fly infection prevalence is often greatest in the wet season or at the end of the “sand fly season”, when few nulliparous females are emerging and physiological sand fly age is greatest (measured by parity) [29]. Variation in the vector’s propensity to blood feed indoors or outdoors may also determine who receives the most infectious bites.

## Mammalian infection reservoirs

Household and near-neighbor clustering supports the assumption that untreated kala-azar cases, long known to be infectious to sand flies [30], comprise the most important infection reservoir fueling transmission during epidemics. Post-kala-azar dermal leishmaniasis (PKDL), a chronic dermatosis that follows apparently successful kala-azar treatment in 5% to 15% of patients in the Indian subcontinent and up to 50% in Sudan, is thought to provide the reservoir that maintains transmission between epidemic cycles [6, 31–33]. PKDL patients are usually not systemically ill, may remain untreated for years, and have been shown to be infectious to sand flies [6, 33–36]. Demonstration of infectiousness requires feeding of laboratory-reared sand flies on the patient (direct xenodiagnosis) or the patient’s blood via a membrane feeder (indirect xenodiagnosis). Because xenodiagnosis is impractical for population-based studies, investigators have sought proxy measures, such as quantitative polymerase chain reaction (qPCR), but the strength and shape of those relationships in different hosts require validation.

In canine leishmaniasis, positive serology or qPCR had high sensitivity (97%–100%) to identify highly infectious dogs but low specificity (13% for serology, 22% for qPCR in ear skin biopsy) [37]. A derived threshold cutoff in ear skin showed sensitivity of 100% to predict highly infectious dogs and specificity of 98% to identify noninfectious ones [37]. Although clinical VL severity was significantly associated with infectiousness, parasite load using the cutoff was a better predictor. These and other canine data also clearly demonstrate that some dogs are “super-spreaders” while others contribute little to transmission: in published xenodiagnosis studies, 15% to 44% of dogs were responsible for >80% of all sand fly infections [38–40]. No such proxies have been validated in human leishmaniasis, although preliminary data from 3 PKDL patients suggest that parasite loads in skin biopsies may provide a proxy for infectiousness [36].

## Asymptomatic infection

Currently, a major question facing VL control efforts in the Indian subcontinent is whether persons with asymptomatic infection are sufficiently infectious to sand flies to constitute an epidemiologically significant infection reservoir [41]. Asymptomatic infections based on seroconversion outnumber clinical disease by 4- to 17-fold, with rising ratios as kala-azar incidence falls [23, 32, 42–45]. If even a subset of asymptomatic individuals are infectious at a very low level, they could still play an important role in transmission, especially when clinical disease incidence is driven to low levels [46, 47]. Failure to address this potential reservoir could preclude interruption of transmission [1].

Recent Indian data show that the median blood parasite load by qPCR is 500-fold higher in kala-azar patients than in asymptomatically infected individuals [48]. Data from the same group confirm that parasite loads in peripheral blood correlate well with those in splenic aspirates [49]. High parasite loads were rare among asymptomatic infections and, when present, indicated individuals with high risk of subsequent development of kala-azar [50]. Antibody titers may also help distinguish asymptomatic infection from “presymptomatic” infection. In a longitudinal study in India, 12% of those with direct agglutination test (DAT) titers >25,600 subsequently developed clinical disease, compared to 1% of those with low titer positive DAT results [44].

In canine leishmaniasis, asymptomatic infected dogs are expected to be less infectious than polysymptomatic dogs through time [38, 51], whereas in naturally infected wildlife hosts, infection is usually benign and associated with relatively low parasite loads and degree of infectiousness (e.g., foxes in Brazil [52] and lagomorphs in Spain [24]). However, asymptomatic animals may have a longer infectious life expectancy than diseased, highly infectious individuals. The canine data reviewed above [37] suggest that qPCR has the most promise as a proxy for xenodiagnosis, but that relationship may vary with parasite tropism and *Leishmania* species [53]. The best specimen type (e.g., peripheral blood or skin biopsy), quantitative technique, and threshold will need to be rigorously validated against xenodiagnosis as the gold standard in each epidemiological setting.

## Influence of sand fly infecting dose on the efficiency of subsequent transmission

In nature, sand flies likely become infected with varying doses of parasites. This initiating dose [54], combined with sand fly immunity, parasite virulence, the sand fly gut microbiota [55–57], environmental conditions, and the blood meal [34], influences parasite development in the gut and subsequent transmission. In particular, the sand fly gut microbiota has recently been shown to heavily influence parasite survival [55–57] and transmission [56]. In an experimental model, transmission via flies infected with varying doses of *L*. *major* parasites was quantified [54]. Higher infecting inocula resulted in greater numbers of parasites per sand fly on day 14 postinfection and higher percentages of metacyclic promastigotes. The percentage of metacyclics was the best predictor of subsequent transmission efficiency to the mammalian host. The bites of high-dose infected flies resulted not only in higher transmission frequencies but also increased disease severity. Temperature, humidity, and oviposition status also significantly influenced transmission efficacy [54]. These observations support the concept, as described for dogs, of “super-spreader” blood meal hosts with high parasite loads resulting in flies with high-dose infections that initiate more severe infections upon subsequent transmission.

## High versus low inocula have differing acute and chronic transmission characteristics

In an analysis of transmission by single sand flies, most infected mice were inoculated with a low dose (<600 parasites); however, for 1 in 4, the inoculum was >1,000 parasites. High-dose transmission resulted from heavy midgut infections, incomplete blood feeding, and transmission of a high percentage of the parasite load from the fly [41]. In a related analysis, low-volume (5-uL) injection of low (100) or high (5,000) doses of sand fly—derived metacyclic promastigotes were inoculated into a restricted dermal site in mice that had been preexposed to sand fly bites. Inoculation of 5,000 parasites into the ear dermis resulted in higher initial parasite loads and more severe acute disease. However, high-dose infections resolved more completely, with a lower lesion size during the chronic phase and a trend towards lower parasite numbers in the skin. Similar observations were published by Lira et. al [58]. Several studies have allowed uninfected flies to feed on the site of primary *L*. *major* infection. As expected, the parasite load in the dermal site of infection directly correlated with the efficiency of transmission from the mammalian host to the vector, with very low parasite loads typically failing to transmit [58–60]. Although the more severe lesions observed at early time points in mice receiving high dose inocula resulted in highly efficient transmission to uninfected flies, these lesions were less efficient at chronic time points. In contrast, lesions initiated with low doses did not result in transmission back to sand flies during early infection but did act as a moderately efficient reservoir during chronic disease [58, 59].

**Fig 2:**
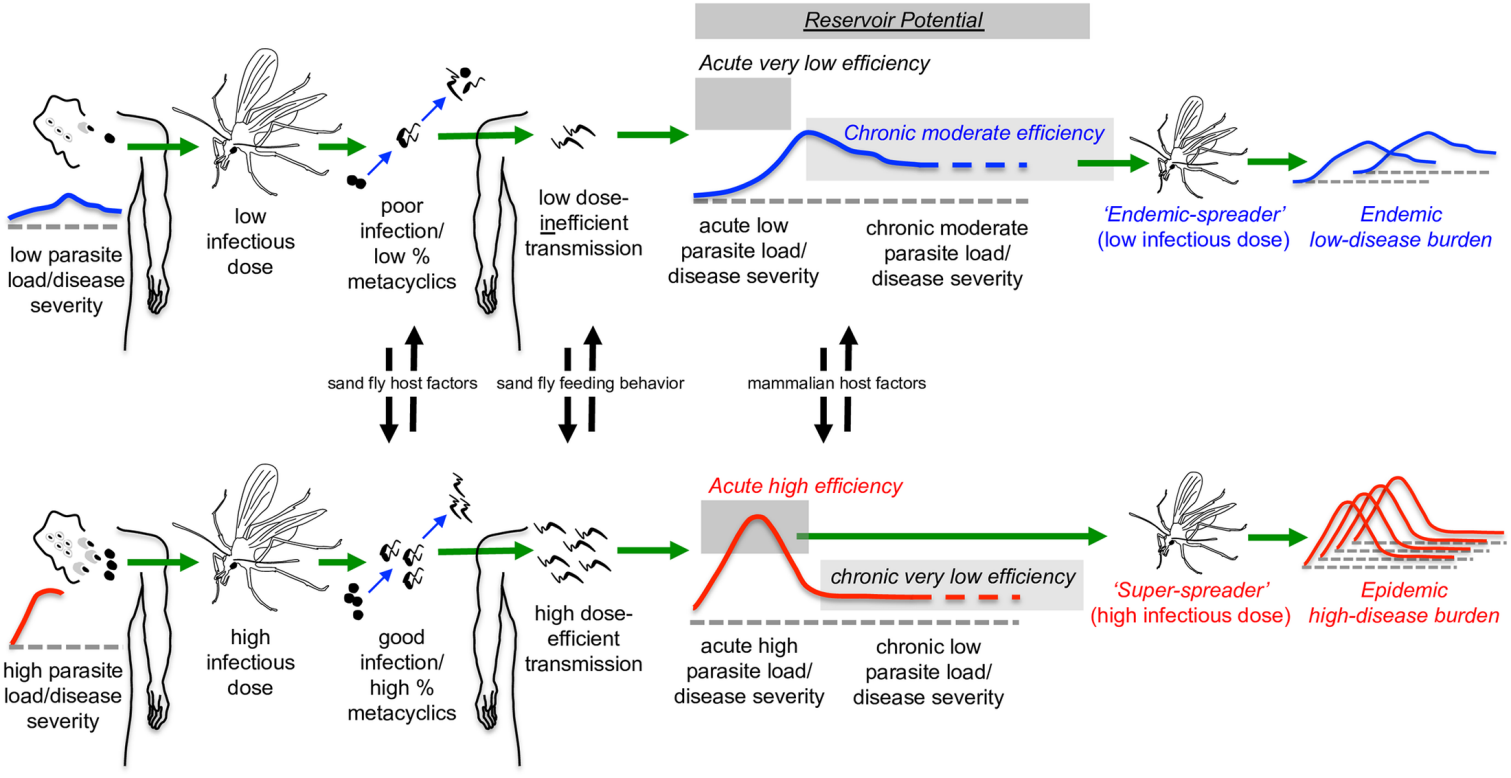
Two modes of sand fly transmission under the influence of dose and the biological inputs that influence them. Flies feeding on mammalian hosts with a high parasite load are infected with a high dose of parasites, generating infections with a high frequency of metacyclic promastigotes that are transmitted to a second mammalian host with high efficiency and in larger numbers, resulting in more severe disease [54]. Higher dose infections in the mammalian host result in more severe acute disease but with more complete resolution and lower parasite loads in the chronic phase. Lower dose infections result in mild acute disease but chronic moderate disease [58, 59]. High acute parasite loads act as highly efficient reservoirs for disease, while low chronic parasite loads are very poor reservoirs for disease, and chronic moderate parasite loads are moderate reservoirs for disease [58–60]. Individuals with high parasite loads are mammalian “super-spreaders” by virtue of their high reservoir potential, while sand flies with high parasite loads are sand fly “super-spreaders” by virtue of their highly efficient transmission of parasites.

These observations suggest 2 non-mutually exclusive modes of transmission (Fig 2). One mode is the acquisition of low numbers of parasites by uninfected sand flies feeding on individuals with low parasite loads and mild or asymptomatic chronic disease. These flies in turn have infections with low parasite numbers and low frequencies of metacyclic promastigotes, and transmit less severe disease. This “mild/asymptomatic” mode of transmission may help explain the maintenance of the parasite in a given population without severe clinical disease. For example, in an investigation in Bhutan, only 1 kala-azar case was detected in a village, yet 35% of the surveyed residents had positive LST results, and the age-prevalence curve strongly suggested chronic low-level transmission over many years [61].

The second mode of transmission occurs when sand flies feed on a heavily infected individual and develop an infection with high parasite numbers and high frequencies of metacyclic promastigotes. When these flies feed on a second host, they transmit a larger number of parasites, causing a more acute and severe disease. On an individual level, the transition from a mild/asymptomatic transmission event to severe/symptomatic transmission may be modulated, for example, when a mammalian host develops severe disease despite a low-dose inoculum due to host factors such as immune status, nutrition, and genetics [62]. In the sand fly host, individual flies infected with a low-dose inoculum may on occasion develop more robust and transmissible infections due to sand fly host factors such as microbiota or sand fly immunity. Alternatively, a poorly infected sand fly may transmit a larger dose of parasites, something that has been shown to occur experimentally, albeit rarely [59]. High-dose transmission by a poorly infected fly is likely related to sand fly feeding behavior, as described below, for the “blocked fly phenotype.”

## Effects of exposure to sand fly saliva on mammalian hosts

Sand flies probe the skin and lacerate the upper dermal capillaries, forming a pool of blood, and continuously inject saliva into the wound to prevent clotting [63, 64]. Sand fly salivary gland homogenate has been shown to exacerbate experimental leishmaniasis in naïve animals when co-inoculated with parasites or confer protection in animals exposed to infected sand fly bites or *Leishmania* plus salivary proteins [65–67]. In experimental models, the most protective salivary proteins induced a delayed type hypersensitivity (DTH) response in skin as early as 6 hours post bite, skewed towards a pro-inflammatory (Th1) phenotype [66, 68]. This focal cellular immunity is thought to act against the earliest stages of infection, reducing parasite survival and ability to initiate disease. Salivary proteins that induce such responses protect against a variety of *Leishmania* species in animal models [63], although the specific mechanism has yet to be fully elucidated. In humans, DTH has also been shown to occur in individuals exposed to uninfected sand flies [69, 70]. Despite this, endemic transmission continues in populations that are frequently bitten by sand flies, suggesting a lack of protection in humans, decay of immune responses between transmission seasons [71], or variable effects of salivary components [72–74].

## *Leishmania* adapt and manipulate their sand fly hosts for efficient transmission

The dose and origin of the infecting parasites influence the course of the infection. Transmission can result from either direct inoculation of parasites in the proboscis or foregut, or regurgitation from a more posterior station in the midgut. As *Leishmania* undergo transformations in the sand fly gut, they produce promastigote secretory gel (PSG) consisting of parasite proteophosphoglycans, including filamentous proteophosphoglycan (fPPG) and secreted acid phosphatases [75–77]. PSG plug formation occurs during metacyclogenesis, when the parasites have succeeded in colonizing the anterior midgut and stomodeal valve [76, 78]. For *L*. *mexicana* in *Lutzomyia longipalpis* [78, 79], the infected midgut can expand to 3 times its original volume, forcing the stomodeal valve permanently open [76]. Damage to the valve may promote reflux of parasites into the skin during blood feeding [80] and *Leishmania* secrete chitinases to further weaken the valve [81]. The combination of PSG and chitinase secretion results in gut distortion and valve dysfunction, causing more persistent feeding attempts and resulting in larger lesions and parasite burdens. In addition, sand flies with the highest number of metacyclic promastigotes have the most fPPG in their midguts and are the most persistent in attempting to feed (the “blocked sand fly” phenotype) [82]. Thus, PSG appears to be the manipulator molecule for *Leishmania*, interfering with blood flow and vector perception of blood intake. Increased blood-feeding persistence is also associated with a higher probability of feeding on multiple hosts in close proximity [76].

Scantily infected flies with enough PSG near the stomodeal valve could disgorge most of their infection in 1 bite and provide exceptions to the relationship between fly infection intensity and onward transmission. In 1 experiment, a single *L*. *major*-infected *Phlebotomus papatasi* with a low infection transmitted 14% of its prefeed load, which was comparable to the high-dose transmitters in the same study [59]. This combination of a low dose of parasites with a high dose of infection-enhancing PSG, exacerbated by modified feeding behavior, may favor acute and severe disease in a host and tip the balance towards the symptomatic/severe form of transmission. If proven, these flies might be considered “super-spreaders” and have epidemiological significance.

PSG has also been shown to enhance *Leishmania* infection in the skin [59] and viscera [83] of mammalian hosts. Interestingly, cutaneous lesions developed at the inoculation site when *L*. *infantum* was coinjected with PSG, suggesting that PSG can promote the survival and persistence of *Leishmania* in skin, irrespective of its cutaneous or visceralizing clinical phenotype, and it may contribute towards the onward transmissibility to other sand flies, as the amastigote dose significantly influences vectorial capacity.

### Implications for transmission and control

The existence of mammalian and sand fly “super-spreaders” provides a biological basis for the spatial and temporal clustering of clinical leishmanial disease. Blood-feeding vectors, including sand flies, are not uniformly distributed within or between susceptible host species [84, 85]. Nonhomogeneous mixing of vectors and hosts usually results in higher transmission rates and greater infection persistence compared to homogeneously mixed populations [84–87]. In nature, infections of wildlife hosts of *Leishmania* are typically subclinical and benign, with varying degrees of tissue tropism, parasite loads, and infectiousness to sand flies, even when hosts live in close association with heavily infected vector populations [53, 88]. Such observations highlight the specificity of host—parasite—vector relationships and the broad spectrum of possible modes of *Leishmania* maintenance and transmission.

Interventions need to have sufficient geographic coverage to include transmission hot spots, especially as current field diagnostic tools do not distinguish highly infectious vectors or hosts from those that are not infectious [38]. To interrupt transmission, specific rapid tests that identify infectiousness are needed. If an intervention suitable for asymptomatically infected individuals were developed, a similar human test would be needed to enable appropriate targeting to those contributing to ongoing transmission. Interventions must be flexible enough to take the dynamics of the disease into account as the leishmaniasis transmission varies spatially and over the course of an epidemic cycle. Interventions that reduce sand fly densities in the absence of elimination could interfere with potential saliva-conferred partial immunity against *Leishmania* [63–70]. Such reductions could also affect vector aggregation dynamics, causing a shift in the attractiveness of sand fly leks from dead-end hosts to humans and animal reservoirs. In turn, this could affect sand fly density-dependent blood-feeding success [89]: incomplete feeding or interrupted probing may lead to multiple bites, promoting transmission within spatially defined host populations [85, 86]. Certainly, sand fly blood-feeding behavior will determine the efficacies of indoor residual spraying, topical insecticides, and bed nets [90, 91]. Alterations in biting behavior affecting the suitability of these methods could be induced by insecticide pressure, as observed in mosquitoes [92, 93], although no such studies have been conducted in sand flies. A deeper understanding of both sand fly and host biology and behavior is therefore essential to ensuring effectiveness of vector interventions and avoiding unintended counterproductive consequences.
